# Effects of physical exercise on Chinese college students’ learning fatigue: the chain mediating role of psychological capital and achievement motivation

**DOI:** 10.3389/fpsyg.2025.1703362

**Published:** 2025-12-03

**Authors:** Hongbo Zhao, Hongxin Huang

**Affiliations:** College of Physical Education, Liaoning Normal University, Dalian, Liaoning, China

**Keywords:** physical exercise, learning fatigue, psychological capital, achievement motivation, college students

## Abstract

**Introduction:**

Learning fatigue has become increasingly prominent among Chinese college students under intense academic competition and educational “involution,” which threatens their mental health and sustainable academic development. Understanding how physical exercise and positive psychological resources may alleviate learning fatigue is therefore of great practical and theoretical significance in the context of Chinese higher education.

**Objective:**

To explore the relationship between physical exercise and learning fatigue among Chinese college students, as well as the relationship between psychological capital and achievement motivation.

**Research method:**

A questionnaire survey was conducted on 502 Chinese college students (54.8% male and 45.2% female) using the Physical Exercise Rating Scale, Learning Fatigue Rating Scale, Psychological Capital Scale, and Achievement Motivation Scale. SPSS 27.0, PROCESS 3.4 plugin, and Bootstrap method were used to analyze the data and test the chain mediation effect of psychological capital and achievement motivation.

**Result:**

The direct impact value of physical exercise on college students’ learning fatigue is −0.008, and the impact values of psychological capital and achievement motivation between physical exercise and college students’ learning fatigue are −0.003 and −0.002, respectively. The chain mediation effect of psychological capital and achievement motivation is significant, with an effect value of −0.001.

**Conclusion:**

(1) Physical exercise can significantly positively predict psychological capital and achievement motivation, and significantly negatively predict learning fatigue; (2) The direct and indirect effects of physical exercise on college students’ learning fatigue are significant. Physical exercise can significantly predict college students’ learning fatigue through the independent mediating effect of psychological capital and achievement motivation, as well as through the chain mediating effect of psychological capital and achievement motivation.

## 1 Introduction

Learning fatigue belongs to negative learning psychology, which refers to students feeling tired of learning due to pressure or lack of interest, resulting in negative attitudes and behaviors (Gong, 2023). Long term learning fatigue manifests as energy depletion, apathy toward peers and behavior, negative academic attitudes, and a lack of academic achievement, which will inevitably lead to a shortage of talent market, waste of teaching resources, and even seriously affect the learning quality and future development of colloege students (Xiao et al., 2021; Wu et al., 2010).

There is a close relationship between physical exercise and learning fatigue. Research has shown that students’ level of physical exercise can affect their level of learning fatigue (Luo et al., 2024; Li, 2024). Students can vent their stress and negative emotions through sports activities, enhance their physical and mental pleasure, help reduce psychological burden, and improve learning fatigue (Zhou and Li, 2024). Physical exercise serves to enhance the psychological capital level of college students, ensure their mental health development, and thus improve learning efficiency (Liu and Jiang, 2024). And the motivation for sports achievement has a significant impact on academic achievement and engagement (Deng et al., 2023), leading to a sense of spiritual satisfaction (Cao et al., 2025).

In China, the learning fatigue experienced by college students is closely related to the broader cultural and educational context. Influenced by Confucian heritage culture, academic achievement and examination performance are often seen as the primary criteria for evaluating young people, which leads families and schools to emphasize hard work, endurance and competition from an early age. At the university level, the phenomenon of “academic involution” has become increasingly prominent, with students feeling that they must invest more and more time and energy in coursework, competitions and certificates merely to maintain their relative position, rather than to gain meaningful learning experiences. This highly competitive and exam-oriented environment can easily result in chronic stress, emotional exhaustion and a sense of meaninglessness in learning, thereby increasing the risk of learning fatigue or academic burnout among Chinese college students. Against this background, it is particularly necessary to explore how physical exercise and positive psychological resources can help alleviate learning fatigue in Chinese higher education.

## 2 Theoretical basis and hypothesis

### 2.1 The relationship between physical exercise and college students’ learning fatigue

The phenomenon of learning fatigue is becoming increasingly prominent among college students (Wang et al., 2025). College students can use physical exercise as a positive way of regulating their body and mind (Xing et al., 2023). According to the stress buffering theory, moderate physical exercise can improve blood circulation in the brain, enhance the excitability and flexibility of the nervous system, thereby helping to improve learning efficiency and reduce fatigue caused by physical fatigue (Mederos et al., 2021; Pan et al., 2016). The theory of emotional regulation suggests that physical exercise provides college students with an outlet to release stress and regulate emotions (Pan et al., 2022). During exercise, students can temporarily relieve themselves from heavy academic workload and alleviate negative emotions such as anxiety and tension (Zhang et al., 2022). This positive emotional experience can enhance students’ confidence and interest in learning, thereby reducing the probability of learning fatigue (Jing et al., 2024). Based on the above analysis, the higher the participation in physical exercise, the lower the level of learning fatigue among college students, thereby improving their learning quality and mental health. Hypothesis H1 is proposed based on this: Physical exercise has a negative predictive effect on learning fatigue among college students.

### 2.2 The mediating role of psychological capital

Psychological capital refers to the sufficient psychological resources provided by individuals in the process of growth and development to cope with negative external environments, and is a key factor for individuals to successfully cope with crises and maintain mental health. The psychological capital of college students is the sum of positive abilities they possess at a special stage of their life development (Du et al., 2025). These positive abilities can be effectively measured and developed to help college students gain self-affirmation and achievement (Nabais et al., 2024). There is a complex correlation between physical exercise and psychological capital (Deng et al., 2023). Studies have found that regular physical exercise can enhance college students’ confidence and improve their psychological capital level (Wei et al., 2024). According to the theory of psychological capital, the pleasure and sense of achievement brought by physical exercise can help cultivate optimistic emotions, alleviate learning pressure, and enhance a sense of hope for learning (Deng et al., 2025). A higher level of psychological capital can effectively reduce the degree of academic fatigue among college students (Zhang and Landicho, 2024). When college students have a high psychological state, they are more willing to actively engage in learning, actively cope with difficulties and challenges in learning, and reduce the fatigue caused by frustration (Wu et al., 2023). Therefore, this study proposes hypothesis H2: Psychological capital plays a mediating role between physical exercise and learning fatigue.

### 2.3 The mediating role of achievement motivation

Achievement motivation, a classic concept first proposed by Atkinson in 1958 and 1964 (Atkinson, 1958; Atkinson, 1964), refers to an individual’s intrinsic drive to strive for success or avoid failure when faced with challenging tasks. Individuals who pursue success tend to view tasks as challenges and therefore choose tasks that are challenging in difficulty and have a high likelihood of success; Individuals who avoid failure tend to view the task as a threat, and they will avoid the occurrence of failure by choosing tasks of lower difficulty. According to self-determination theory, physical exercise can promote the secretion of neurotransmitters in the brain, enhance an individual’s self-confidence and self-efficacy, thereby increasing their achievement motivation (Tao et al., 2023), and they can overcome difficulties during exercise to gain a sense of achievement (Cao et al., 2025). When college students’ achievement motivation increases, they are more willing to challenge themselves and pursue higher academic achievements (Li et al., 2024). The higher the positive achievement motivation, the lower the level of academic burnout among college students (Alivernini and Lucidi, 2011), a pattern that has also been confirmed in recent studies linking achievement motivation to academic burnout and engagement (Li et al., 2023). Based on this, this study proposes hypothesis H3: Achievement motivation mediates the relationship between physical exercise and learning fatigue.

### 2.4 Chain mediation of psychological capital and achievement motivation

**Figure 1:**
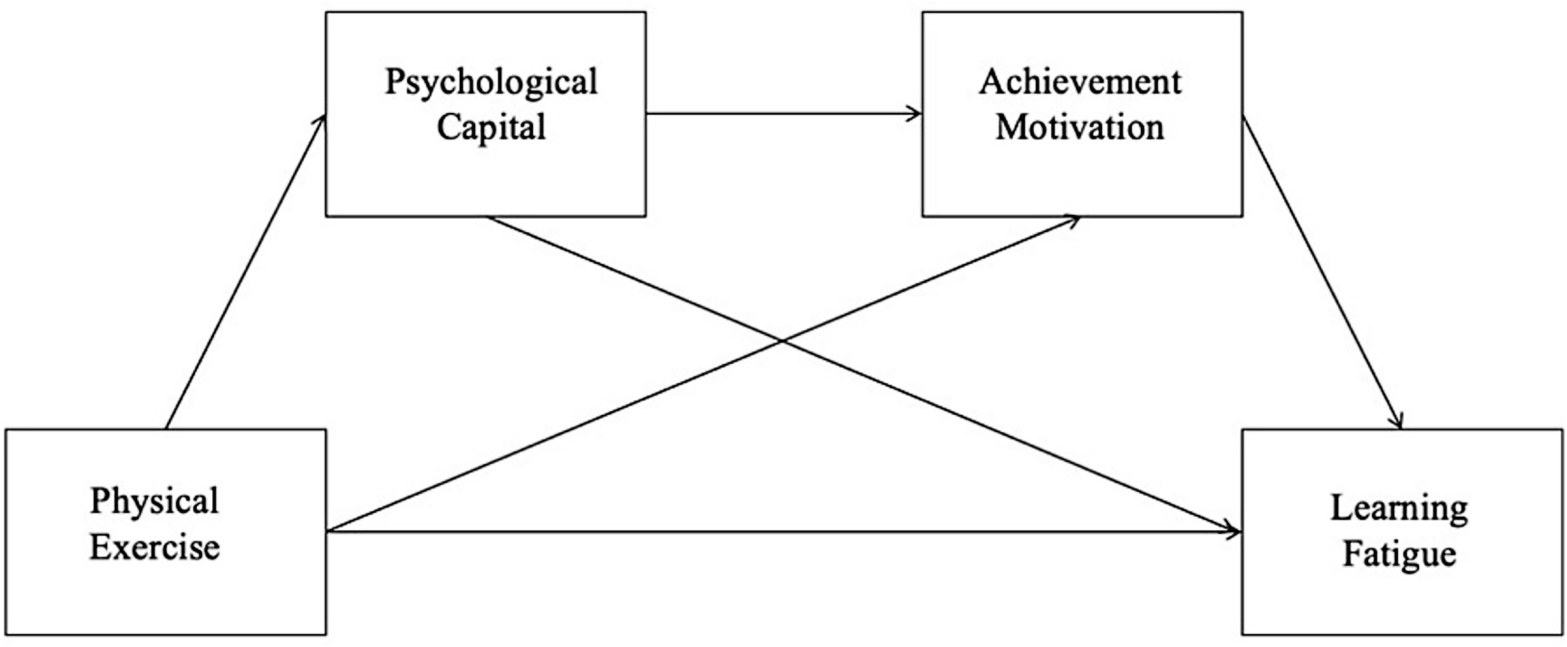
Conceptual model diagram. This figure was created by the authors for the present study.

Psychological capital, as an individual’s positive psychological state, affects the learning status of college students (Wu et al., 2022) and is also influenced by physical exercise (Zou et al., 2023). According to social cognitive theory, in a physical exercise environment, individuals’ psychological capital and achievement motivation are influenced by social interactions, successful experiences, and role models, thereby enhancing (Yan et al., 2024; Messina et al., 2024). There is a strong correlation between the psychological capital and achievement motivation of college students (Sarwar and Rahman, 2017). Achievement motivation is the internal driving force for individuals to pursue success and avoid failure, and it plays a key role in their behavioral choices and psychological experiences (Zhao et al., 2025). The improvement of psychological capital will stimulate individuals’ achievement motivation, making them more proactive in facing learning tasks and thereby reducing learning fatigue. Therefore, the chain mediation hypothesis H4 is proposed: psychological capital and achievement motivation play a chain mediation role between physical exercise and learning. Based on previous theories and studies, the hypothesized model shown in Figure 1.

In the past 5 years, an increasing number of studies have examined how physical activity relates to academic or learning burnout among college students, often focusing on single mediating variables such as self-efficacy, resilience, mindfulness, self-esteem, psychological resilience, or positive emotions (Chen et al., 2022; Tian and Yang, 2024; Xu, 2025; Gong et al., 2023). Recent research has shown that physical exercise is negatively associated with academic burnout and that this association can be explained by serial mediating paths including self-efficacy and resilience, mindfulness and self-esteem, or self-control and coping styles (Jin et al., 2024; Song et al., 2025). Other studies have highlighted the importance of psychological capital and related constructs in understanding learning burnout among college students, and some work has also linked achievement motivation to students’ engagement and burnout (Zhong et al., 2025; Wang and Jiang, 2025). However, most of these studies have not specifically targeted learning fatigue in Chinese college students, and very few have simultaneously tested psychological capital and achievement motivation as chain mediators in one integrated model. To address these gaps, the present study focuses on Chinese college students’ learning fatigue and constructs a chain mediation model in which psychological capital and achievement motivation jointly transmit the effect of physical exercise to learning fatigue, thereby extending previous mediation models and providing new empirical evidence from the context of Chinese higher education.

## 3 Research participants and methods

### 3.1 Participants and sampling

A total sampling method was used to select 550 college students from Liaoning Normal University in Dalian City, Liaoning Province, China, to conduct a questionnaire survey, and the consent of the school, parents and the subjects themselves was obtained before the test. Inclusion criteria: (1) clear consciousness, able to successfully complete the questionnaire, and no history of mental illness; (2) Voluntarily participate in the investigation of this study; (3) In good health, not included in the clinical disease. Among the recovered questionnaires, questionnaires with one of the following questions were considered invalid questionnaires: (1) inconsistent responses to reverse-scoring questions; (2) The answer time is too short; (3) There is an obvious repetition pattern in answer selection, such as 1, 2, 3, 1, 2, 3. Among them, 13 questionnaires with inconsistencies before and after answering reverse scoring questions, 26 questionnaires with too short answering time, and 9 questionnaires with regular answers were excluded. In the end, after excluding the invalid questionnaires, there were 502 valid questionnaires left, with an effective rate of 91.27%. Among them, 275 were male (54.8%) and 227 were female (45.2%).

### 3.2 Research methodology

#### 3.2.1 Physical exercise scale

Adopting the revised Physical Exercise Rating Scale (PARS-3) developed by Hashimoto Kimio and revised for Chinese college students by Liang (1994). The amount of physical exercise evaluated based on three dimensions: intensity, frequency, and duration of participation. Each index was scored on a Likert scale of 5 points, and the amount of physical exercise activity = exercise intensity× duration× exercise frequency, and the higher the score, the higher the degree of exercise participation with a maximum score of 100 and a minimum score of 0. There is one item in terms of physical exercise intensity, 1 = light exercise (e.g., walking) 2 = light intensity exercise (e.g., tai chi) 3 = intensity from participating in physical exercise 4 = high-intensity exercise (e.g., basketball) 5 = high-intensity sustained exercise (e.g., running). In terms of the time of physical exercise, there is one item, 1 = less than 10 min, 2 = 11 min to 20 min, 3 = 21 min to 30 min, 4 = 31 min to 59 min, and 5 = 60 min. There is one item in terms of the frequency of physical activity, 1 = once or twice a month 2 = one 4 or 5 times 3 = 1 to 2 times a week 4 = 3 to 5 times a week 5 = about once a day. The Cronbach’s alpha coefficient for internal consistency of the scale in this study is 0.73.

#### 3.2.2 Psychological capital scale

This study used the Positive Psychological Capital Questionnaire (PPQ) developed by Zhang et al. (2010), Fu et al. (2014). Since the concept of psychological capital was proposed, numerous researchers have explored its content. Zhang Kuo et al. summarized research both domestically and internationally and found that the four factor structure of psychological capital has been widely accepted by scholars (Du et al., 2025; Nabais et al., 2024). Therefore, they also adopted a questionnaire structure consisting of four dimensions and 26 items. Using a Likert 1–5 five point rating, the higher the score, the higher the level of psychological capital. Example items include “I am full of confidence in my ability to achieve my goals” and “When I encounter difficulties in my studies, I can usually find a way to overcome them.” The Cronbach’s alpha of the psychological capital scale in this study was 0.92.

#### 3.2.3 Achievement motivation scale

This study used the Chinese version of the Achievement Motivation Scale translated and revised by Ye and Kunt (1992). The revised scale consists of 30 items, including two dimensions of pursuing success and avoiding failure, each with 15 items, and has been widely applied by many experts and scholars (Han et al., 2022; Huang, 2023). Using a Likert 1–5 five point rating, the higher the score, the higher the level of achievement motivation. Example items include “I like to take on challenging learning tasks” and “I feel satisfied when I can do better than my classmates.” The Cronbach’s alpha of the achievement motivation scale in this study was 0.93.

#### 3.2.4 Learning fatigue scale

Adopting the “College Student Academic Burnout Scale” developed by Lian Rong et al. in 2005 (Kwok et al., 2015). The entire questionnaire consists of 20 items, including 3 dimensions: low mood, inappropriate behavior, and low sense of achievement. The dimension of low mood (such as being able to handle emotional problems calmly while studying) includes 8 items, the dimension of inappropriate behavior (such as feeling that the knowledge learned is useless) includes 6 items, and the dimension of low sense of achievement (such as easily mastering professional knowledge for me) includes 6 items. Using the Likert 5-point rating system, the higher the score, the higher the level of learning fatigue. The Cronbach’s alpha coefficient of the scale in this study is 0.90, indicating high reliability.

### 3.3 Data statistics and analysis

This study used SPSS 27.0 statistical software for data analysis. First, reliability was tested using Cronbach’s alpha test and common method bias after data collection was tested using Harman’s one-way test. Second, after importing the data into SPSS, demographic analysis was performed using descriptive statistics. Pearson’s correlation coefficient was used to analyze the correlation between physical exercise, psychological capital and achievement motivation and learning fatigue. Multiple regression analyses were conducted using Model 6 in the SPSS PROCESS macro program developed by Hayes. The Bootstrap test was used to evaluate the significance level of the mediating effect, and then the study was analyzed. Among them, the Bootstrap method test set a sample size of 5,000 with a confidence interval of 95%, where the effect was significant if the confidence interval did not include zero, and vice versa. Of the 550 questionnaires distributed, 502 valid questionnaires were retained after excluding cases with missing values on key study variables or obvious response patterns. Because all retained questionnaires had complete data on the main variables, the analyses were conducted using listwise deletion and no imputation for missing values was required.

## 4 Research results

### 4.1 Common method bias test

To avoid the impact of common method bias on research results, necessary controls were implemented during the actual testing process, such as requiring participants to answer anonymously and using reverse scoring for some items. Using Harman’s single factor test for common method bias testing, the results showed that there were 10 factors with eigenvalues greater than 1, and the first factor explained 21% of the variance, which was less than the critical value of 40%. Therefore, it is believed that there is no significant common method bias in this study.

### 4.2 Descriptive statistics and correlation analysis of each variable

Using independent sample t-test, analyze the differences in physical exercise, psychological capital, achievement motivation, and learning fatigue among college students of different genders and grades. The results showed that boys were superior to girls in terms of physical exercise, psychological capital, and achievement motivation, showing significant differences. The physical exercise, psychological capital, and achievement motivation of second year college students are higher than those of other grades, showing significant differences. Learning fatigue is highest among third grade college students, showing a significant difference (Table 1).

**Table 1:** Sample size demographic information and independent sample *t* test.

Dependent variable	Independent variable	*N* = 502	M ± SD	t	*p*
Physical exercise	Gender	Male(275)	36.593 ± 28.477	8.135	<0.001
		Female(227)	18.401 ± 19.810		
	Grade	Freshman (116)	27.164 ± 25.490	2.958	0.032
		Sophomore(118)	34.212 ± 27.687		
		Junior(138)	24.601 ± 26.572		
		Senior (130)	28.130 ± 25.630		
Psychological capital	Gender	Male(275)	3.174 ± 0.729	2.462	0.014
		Female(227)	3.013 ± 0.731		
	Grade	Freshman (116)	3.097 ± 0.714	11.828	<0.001
		Sophomore(118)	3.411 ± 0.609		
		Junior(138)	2.888 ± 0.728		
		Senior (130)	3.050 ± 0.770		
Achievement motivation	Gender	Male(275)	3.110 ± 0.717	2.380	0.018
		Female(227)	2.957 ± 0.728		
	Grade	Freshman (116)	2.986 ± 0.727	5.723	<0.001
		Sophomore(118)	3.256 ± 0.667		
		Junior(138)	2.894 ± 0.710		
		Senior (130)	3.050 ± 0.753		
Learning fatigue	Gender	Male(275)	3.052 ± 0.722	−3.205	<0.001
		Female(227)	3.263 ± 0.741		
	Grade	Freshman (116)	3.154 ± 0.759	3.191	0.023
		Sophomore(118)	2.984 ± 0.676		
		Junior(138)	3.266 ± 0.772		
		Senior (130)	3.167 ± 0.719		

As shown in Table 2, Pearson correlation analysis was performed on physical exercise, psychological capital, achievement motivation and learning burnout using gender and grade as control variables. The results showed that there was a significant correlation between the study variables, and there was a significant positive correlation.

**Table 2:** Correlation analysis between variables.

	Physical exercise	Psychological capital	Achievement motivation	Learning fatigue	Gender	Grade
Physical exercise	1					
Psychological capital	0.358**	1				
Achievement motivation	0.395**	0.437**	1			
Learning fatigue	−0.483**	−0.502**	−0.482**	1		
Gender	−0.342	−0.109	−0.106	0.142	1	
Grade	−0.030	−0.104	−0.028	0.050	0.680	1
M ± SD	28.36 ± 26.51	3.10 ± 0.73	3.04 ± 0.72	3.14 ± 0.73	1.45 ± 0.49	2.56 ± 1.10

*N* = 502. *p < 0.05, **p < 0.01, ***p < 0.001.

### 4.3 Test of intermediate effect

As shown in Table 3, Model 6 of the SPSS macroprogram developed by Hayes is used to test the chain mediation effect. Gender and grade were used as control variables to test the mediating role of psychological capital and achievement motivation in the relationship between physical exercise and college students’ psychological capital. The results showed that physical exercise had a significant positive predictive effect on psychological capital (=0.336, *p* < 0.001) and achievement motivation (=0.273, *p* < 0.001), psychological capital had a significant positive predictive effect on achievement motivation (=0.325, *p* < 0.001), a significant negative predictive effect on learning fatigue (= − 0.284, *p* < 0.001), and achievement motivation had a significant negative predictive effect on learning fatigue (= − 0.232, *p* < 0.001). Psychological capital and achievement motivation were included in the structural equation, and physical exercise had a significant negative predictive effect on college students’ learning fatigue (= − 0.280, *p* < 0.001).

**Table 3:** Regression analysis between variables.

Result variable	Variable of prediction	Fit the indicator			Coefficient significance	
		R	R^2^	F	*β*	t
Psychological capital	Gender	0.157	0.150	23.103**	0.002	0.052
	Grade				−0.226	−3.990***
	Physical exercise				0.336	7.566***
Achievement motivation	Gender	0.265	0.258	35.846**	0.019	0.471
	Grade				−0.022	−1.337
	Physical exercise				0.273	6.229***
	Psychological capital				0.325	7.767***
Learning fatigue	Gender	0.401	0.397	111.106**	−0.006	−0.175
	Grade				0.084	1.730***
	Physical exercise				−0.280	−6.848***
	Psychological capital				−0.284	−7.128***
	Achievement motivation				−0.232	−5.741***

*N* = 502. *p < 0.05, **p < 0.01, ***p < 0.001.

### 4.4 Chain mediation model test

**Figure 2:**
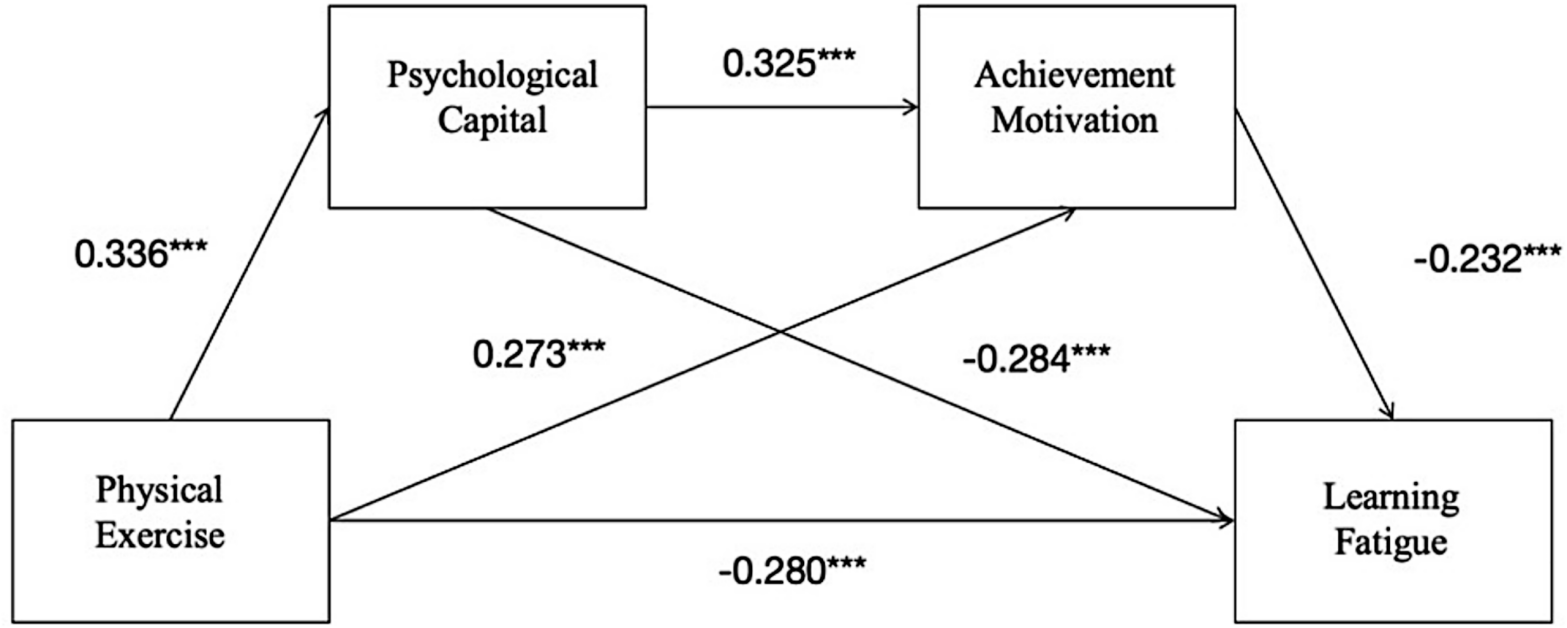
A mediating model of physical exercise affecting college students’ learning fatigue (***p < 0.001). Standardized path coefficients are based on data from 502 Chinese college students collected in the present study.

The results of the path coefficients are shown in Figure 2. The bias-corrected percentile Bootstrap method was used to test the mediating effect, and Standardized path coefficients, effect sizes and their 95% confidence intervals are reported in Table 4. The total mediating effect of psychological capital and achievement motivation between physical exercise and college students’ learning fatigue was −0.006, and the ratio of the mediating effect to the total effect was 42.86%. The 95% confidence interval did not contain zero, indicating that the total mediating effect between physical activity and college students’ learning fatigue was significant. The overall mediating effect consisted of three paths: Ind1: the mediating effect of psychological capital on the relationship between physical exercise and college students’ learning fatigue, with a mediating effect value of −0.003 and a mediating effect ratio of 21.43% to the total effect. The 95% confidence interval does not contain zero, indicating that the mediating effect of psychological capital is significant. Ind 2: The mediating effect of achievement motivation on the relationship between physical exercise and college students’ learning fatigue was −0.002, and the ratio of the mediating effect to the total effect was 14.29%. The 95% confidence interval did not contain zero, indicating that the mediating effect of achievement motivation was significant. Thirdly, the chain mediating effect of psychological capital and achievement motivation between physical exercise and learning fatigue was −0.001, and the ratio of the mediating effect to the total effect was 7.14%. The 95% confidence interval did not contain zero, indicating that the chain mediation effect was significant. The hypothesis of the study was confirmed. Instead: The above results show that the three indirect effects have reached a significant level, and it is assumed that H1, H2, H3, and H4 are true.

**Table 4:** Proportion of the mediating effect.

Influence path	Effect size	Boot SE	95% confidence interval		Proportion
			BootLLCI	BootULCI	
Total effect	−0.014	0.001	−0.016	−0.011	100%
Direct effect	−0.008	0.001	−0.10	−0.006	57.14%
Ind1	−0.003	0.001	−0.004	−0.002	21.43%
Ind2	−0.002	0.000	−0.003	−0.001	14.29%
Ind3	−0.001	0.000	−0.001	−0.000	7.14%
Total indirect effect	−0.006	0.001	−0.007	−0.004	42.86%

## 5 Discussion

In order to explore the internal mechanism of physical exercise on college students’ learning fatigue, our study constructed a chain mediation model and analyzed the chain mediating effect of psychological capital and achievement motivation on college students’ learning fatigue. This study not only helps to reveal the impact of physical exercise on learning fatigue and its internal mechanism, but also provides empirical evidence and suggestions for college students’ learning fatigue.

### 5.1 Relationship between physical exercise and college students’ learning fatigue

The results of this study confirmed that physical exercise was significantly negatively correlated with college students’ study fatigue, suggesting that physical activity was a positive factor affecting college students’ study fatigue, that is, the higher the amount of physical exercise, the higher the level of learning fatigue the lower the level of learning fatigue, which was consistent with the results of previous studies (Jing et al., 2024). The amount of physical activity is generally higher and the learning fatigue level is lower in male college students than in female students, which may be partly related to gender differences in physical fitness and exercise participation (Gan et al., 2001). Recent surveys also show that male college students tend to engage more in regular physical activity than females (Zhang et al., 2023). The amount of exercise in lower-grade college students is higher than that in senior grades, while the burnout value of junior college students is also the highest, which may be related to the increasing employment and examination pressure in higher grades (Gao, 2013). Long-term and sustained physical activity can have a positive effect on both positive and negative emotions (Wang et al., 2024). At the same time, physical activity can provide a place for students to express and vent their bad emotions, thereby reducing psychological pressure, relieving their tension, and facilitating the recovery of emotional resources, thereby reducing learning fatigue and improving and promoting students’ physical and mental health (Zhang, 2011).

### 5.2 Mediating role of psychological capital

The results of this study found that psychological capital plays a mediating role in the relationship between physical activity and college students’ learning fatigue, which is consistent with the results of previous studies (Wu et al., 2023). Psychological capital is an important protective factor for college students’ emotional and academic problems (Atkinson, 1964; Tao et al., 2023; Huang, 2017). College students with high psychological capital are more likely to maintain positive learning attitudes and emotional experiences, which helps reduce the level of learning fatigue (Lehto et al., 2012; Lupu and Özcan, 2014). Physical activity is also an important factor influencing learning fatigue (Lupu and Özcan, 2014), and recent chain mediation studies have shown that exercise can reduce academic burnout by enhancing resilience, self-esteem and other positive psychological resources (Atkinson, 1958; Alivernini and Lucidi, 2011). When individuals feel supported by their peers and improve their own psychological capital, learning fatigue is significantly reduced (Deng et al., 2025).

### 5.3 Mediating role of achievement motivation

The results of this study found that achievement motivation plays a mediating role in the relationship between physical exercise and college students’ learning fatigue, which is consistent with previous studies (Li et al., 2023). During physical activity, college students can repeatedly experience success by setting exercise goals and increasing training intensity, and this positive experience is closely related to the motivation to pursue success (Ma, 2011). When individuals have a high achievement motivation, they are more likely to regard learning as a challenge rather than a burden, and actively engage in learning tasks, which helps reduce learning fatigue (Li et al., 2023; Li, 2016). Conversely, students with low achievement motivation are more likely to feel tired and bored in their studies (Liu, 2020).

### 5.4 The mediating chain effect of physical exercise and college student’ learning fatigue

The results of the chain mediation test showed that psychological capital and achievement motivation had a chain mediating effect on the relationship between physical exercise and college students’ learning fatigue. Psychological capital is an important factor influencing achievement motivation (Liang, 2014), and empirical studies have shown that college students with higher levels of psychological capital tend to report stronger achievement motivation and lower academic burnout (Atkinson, 1964; Tao et al., 2023; Zou and Wei, 2016). When psychological capital is low, students’ self-efficacy decreases and they are more likely to avoid learning tasks and experience learning fatigue (Mou and Huang, 2025; You, 2016). Therefore, long-term and regular physical exercise can improve the psychological capital of college students and enhance their achievement motivation (Wu et al., 2023; Atkinson, 1958; Alivernini and Lucidi, 2011; Xiong et al., 2017), thereby reducing the level of learning fatigue.

## 6 Conclusion

When the mediating variables are released, the direct and indirect effects of physical exercise on college students’ learning fatigue are significant, which can be mediated by the mediating effect of psychological capital and achievement motivation, and the chain mediation effect of psychological capital and achievement motivation can be used as the mediating effect. The findings of this study have several practical implications for Chinese higher education. First, in the context of Confucian culture and academic involution, where examination performance and rankings are still the main criteria for evaluating students, our results suggest that physical exercise can serve as a low-cost and culturally acceptable way to alleviate learning fatigue. Universities should therefore guarantee sufficient time and resources for regular physical activity, and integrate physical education courses with stress management and mental health promotion. Second, because psychological capital and achievement motivation play chain mediating roles between physical exercise and learning fatigue, intervention programs that combine physical exercise with training in optimism, resilience, self-efficacy and goal setting may be particularly effective for Chinese college students. Third, at the system level, the results support recent educational reforms that aim to reduce excessive academic pressure and “involution” by diversifying evaluation criteria and valuing students’ physical and mental health. Policymakers and university administrators can use indicators such as physical activity, psychological capital and learning fatigue to design early warning systems and targeted support services for students at risk of academic burnout.

## 7 Limitations and future prospects

First, this study adopted a cross-sectional design and recruited participants from a single university, which limits causal inference and the generalizability of the findings to other regions or types of institutions; future research should use longitudinal or experimental designs and include more diverse samples from multiple universities with better control of potential confounders such as socioeconomic status, academic major and prior physical activity. Secondly, this paper mainly belongs to the cross-sectional study, and the college students are used as the survey group, which may limit the effectiveness of causal inference, and it is necessary to expand the sampling scope and sample size in the future, and adopt a combination of horizontal and vertical research methods to explore the causal relationship. Finally, experimental studies can be used to explore the relationship between physical exercise and college students’ learning fatigue and various variables. It is hoped that this study can provide a reference for college students to obtain a higher sense of security in physical exercise, and provide a basis for schools and parents to understand the content of college students’ ability of learning.

## Data Availability

The raw data supporting the conclusions of this article will be made available by the authors, without undue reservation.
